# Serum Calcium Concentration Is Associated with Bone Mineral Density and Synonymous Variants in the *RYR1* Gene in a Mexican-Mestizo Population

**DOI:** 10.3390/medsci13040324

**Published:** 2025-12-17

**Authors:** Tania V. López-Pérez, Rogelio F. Jiménez-Ortega, Armando Cruz-Rangel, Diana I. Aparicio-Bautista, Juan C. Fernández-López, Adriana Becerra-Cervera, Juan P. Reyes-Grajeda, Jorge Salmerón, Alberto Hidalgo-Bravo, Berenice Rivera-Paredez, Rafael Velázquez-Cruz

**Affiliations:** 1 Laboratorio de Genómica del Metabolismo Óseo, Instituto Nacional de Medicina Genómica (INMEGEN), Mexico City 14610, Mexico; tperez@inmegen.edu.mx (T.V.L.-P.); daparicio@inmegen.gob.mx (D.I.A.-B.); abecerra@inmegen.edu.mx (A.B.-C.); 2 Secretaría de Ciencia, Humanidades, Tecnología e Innovación (SECIHTI), Mexico City 03940, Mexico; 3 Servicio de Medicina Genómica, Instituto Nacional de Rehabilitación Luis Guillermo Ibarra Ibarra (INRLGII), Mexico City 14389, Mexico; rfjimenez@inr.gob.mx (R.F.J.-O.); dr_genetica@yahoo.com (A.H.-B.); 4 Laboratorio de Estructura de Proteínas, Instituto Nacional de Medicina Genómica (INMEGEN), Mexico City 14610, Mexico; acruz@inmegen.gob.mx (A.C.-R.); jreyes@inmegen.gob.mx (J.P.R.-G.); 5 Departamento de Genómica Computacional, Instituto Nacional de Medicina Genómica (INMEGEN), Mexico City 14610, Mexico; jfernandez@inmegen.gob.mx; 6 Centro de Investigación en Políticas, Población y Salud (CIPPS), Facultad de Medicina-Universidad Nacional Autónoma de México (UNAM), Mexico City 04510, Mexico; jorge.salmec@gmail.com

**Keywords:** serum calcium, Ryanodine Receptor1, calcium channels, bone mineral density, synonymous variants

## Abstract

Background/Objectives: Serum calcium concentrations have been associated with bone mineral density (BMD), but results seem to depend on sex. Genetic variants in the Ryanodine Receptor1 (*RYR1*) gene have been previously associated with low BMD in postmenopausal women. Serum RYR1 concentration was found to be higher in osteopenia and osteoporosis groups. The function and biological relevance of RYR1 in bone remodeling remains unknown. This cross-sectional study explored the relationship between serum calcium concentrations, BMD, and genetic variants in *RYR1* in a Mexican-mestizo population. Methods: Serum samples from 966 participants were obtained from the third measurement of the Health Workers Cohort Study (HWCS) 2017–2019, conducted by the Mexican Social Security Institute (IMSS). All participants included in this study were of Mexican Mestizo origin and had data on BMD. We measured ionized calcium and genotyped the genetic variants rs2288888 (g.38455542G>A) and rs11083462 (g.38469040C>T) of the *RYR1* gene. BMD of the total hip, lumbar spine, and femoral neck was measured using a Lunar DPX NT DEXA device. Results: Our results show that elevated serum calcium concentrations in females are associated with lower BMD at the hip and femoral neck. In contrast, higher calcium concentrations in males were associated with greater total hip BMD. In our study, the variants rs2288888 and rs11083462 were associated with higher serum calcium concentrations (under-adjusted and unadjusted data) in males but not females. Conclusions: Serum calcium levels are associated with BMD, depending on sex. The *RYR1* gene variants rs2288888 and rs11083462 may have a protective effect in men.

## 1. Introduction

Calcium is essential for heart function, bone integrity and muscle contraction and participates as a second messenger in of different signaling pathways. After calcium intake, the gastrointestinal system absorbs it through the action of the parathyroid hormone (PTH) and 1,25-dihydroxyvitamin D, which, together with calcitonin, are responsible for serum calcium regulation. Furthermore, calcium is crucial for bone matrix mineralization, as osteoclasts and osteoblasts rely on calcium signals to regulate their differentiation and activity [1].

The association between serum calcium and bone mineral density (BMD) has been studied in adolescents [2] and older adults [3], the data show differences related to sex and age [4,5]. This becomes relevant because calcium and vitamin D supplements are consumed to prevent osteoporosis, especially at older ages. Osteoporosis (OP) is a disease in which bone remodeling homeostasis is disrupted, leading to progressively reduced bone mineralization and increased bone fragility. Early diagnosis of OP is essential to reduce healthcare burden and improve patients’ quality of life [6,7]. Identifying biomarkers to assess bone health is essential for prevention, timely diagnosis, treatment, and follow-up. Proteomic research has identified proteins differentially expressed in women with normal and low BMD [8,9]. Based on their role on bone metabolism and a thorough analysis of protein interactions, we previously identified a panel of 12 candidate biomarkers in serum samples from postmenopausal Mexican women. Among these 12 candidate biomarkers, Ryanodine Receptor 1 (RYR1) was found to be upregulated in the osteopenia (OS) and OP groups [10].

The ryanodine receptors are located at the endoplasmic/sarcoplasmic reticulum membrane. They are homotetrameric calcium channels, and their function is to mediate the release of calcium (Ca^2+^) from the intracellular compartments to produce cellular responses. They intervene in processes such as contraction of cardiac and skeletal muscle, and to induce prolonged signaling in neurons [11]. There are three isoforms in mammals: RYR1 is prominent at skeletal muscle and brain, RYR2 in cardiac muscle and β-cells and RYR3 is ubiquitously expressed [11,12]. Voltage activates transepithelial dihydropyridine receptors (DHPRs), which interact with RYR proteins to initiate the release of Ca^2+^ stored in the endoplasmic reticulum of skeletal and cardiac muscle cells [12].

The RYR channels have been broadly studied in skeletal muscle and cardiac channelopathies. Studies have shown that genetic variants in RYR channels influence channel activity, either by favoring their sensitivity [13,14] or promoting calcium leakage [15], leading to heart problems [16,17] and skeletal muscle disorders [18]. However, these variants have only been studied in these disorders. Two previous studies have reported that individuals with RYR1-related disorders (arrhythmias and myopathies) have bone problems such as scoliosis [19] and osteoporosis at an early age [20]. The precise function and biological relevance of RYR channels in bone remodeling remains unknown, although it has been suggested that RYR channels may be associated with osteoclast differentiation or function [12,21]. In a previous genome-wide association study (GWAS), carried out on a cohort of Mexican-Mestizo postmenopausal women, a subsample of the Health Workers Cohort Study (HWCS), we identified genetic variants located in the *RYR1* gene, associated with BMD at the femoral neck [22]. In this cross-sectional study, we explored the relationship between serum calcium, BMD, and genetic variants in *RYR1* in an admixed Mexican-mestizo population.

## 2. Materials and Methods

### 2.1. Study Population

Serum samples from 966 participants were obtained from the third measurement of the Health Workers Cohort Study (HWCS) 2017–2019, conducted by the Mexican Social Security Institute (IMSS) [23]. All participants included in this study were of Mexican Mestizo origin and had data on BMD. The study protocol was approved by the Ethics Committees of the Mexican Social Security Institute (12CEI 09 006 14) and the National Institute of Genomic Medicine (CEI/2025/19). Written informed consent was obtained from all participants, and all procedures were performed in accordance with the Declaration of Helsinki (13/LO/0078). The study population resides in Cuernavaca, Morelos, an area of Central México, where the predominant indigenous ancestry is from the Nahua group, which has historically been settled in this region [24].

### 2.2. BMD Measurement

BMD (g/cm^2^) at the total hip, lumbar spine, and femoral neck was measured using a Lunar DPX NT dual-energy X-ray absorptiometry (DXA) device (Lunar Radiation Corp., Madison, WI, USA). According to World Health Organization criteria, low BMD was defined as a T-score below −1.0 [25].

### 2.3. Serum Sample Preparation

Blood samples were collected from all participants after an 8-h fasting period to ensure precise metabolic measurements. The samples were centrifuged at 2643× *g* for 15 min, and serum was isolated. Serum samples were aliquoted and stored at −80 °C until they were required for analysis.

### 2.4. Ionized Calcium Serum Determination

Serum calcium determination was performed using the calcium assay, Cat 3L79-42 (Abbott Laboratories, Abbott Park, IL, USA) following the manufacturer’s instructions using the ARCHITECT cSystems equipment.

### 2.5. Single Nucleotide Variant Selection and Genotyping

Based on previous study [22], two genetic variants were selected for analysis: rs11083462 (g.38469040C>T) and rs2288888 (g.38455542G>A) on the *RYR1* gene. Genomic DNA was extracted from peripheral blood leukocytes using the PUREGENE DNA Blood Kit (QIAGEN Systems Inc., Valencia, CA, USA) according to the manufacturer’s protocol. Genotyping was performed using predesigned TaqMan SNP Genotyping assays (Applied Biosystems, Foster City, CA, USA) in a QuanStudio 7 Flex Real-Time PCR System (Applied Biosystems, Waltham, MA, USA). Data were analyzed using Sequence Detection System (SDS) software, version 2.2.1.

### 2.6. Localization of Genetic Variants in the Homotetrameric Structure of RYR1

The location of the variants rs11083462 (g.38469040C>T) and rs2288888 (g.38455542G>A) of the *RYR1* gene was determined using the Ensembl genome browser database. Subsequently, the translation of the mRNA was performed using the Expasy Translation Tool of the Swiss Institute of Bioinformatics, which locates the amino acid encoded by the codon where the variants lie. We previously identified these variants as synonymous. However, to determine whether these variants are located within a core domain associated with the function of the RYR1 channel, a modeling approach was employed to predict their location within the homotetrameric structure of RYR1. The spatial positioning of the amino acids encoded by the rs11083462 and rs2288888 in the homotetrameric structure of RYR1 was created using UCSF ChimeraX with the 7TDG PDB structure as a model [26].

### 2.7. Statistical Analysis

Data from the study population are shown as medians for quantitative variables as well as absolute and relative frequencies. Hardy-Weinberg equilibrium was performed for each genetic variant using the χ^2^ test. Linear and logistic regression analyses were conducted to evaluate the association between BMD, serum calcium concentrations, and genotype, as appropriate. Genetic models tested included codominant, additive, recessive, and dominant. Models on BMD were adjusted for age (years), alcohol consumption, smoking, calcium intake, calcium supplement intake, physical activity, body mass index, hormone replacement therapy (HRT) (women only), serum vitamin D concentrations, and albumin concentrations. To evaluate the effect of serum calcium on BMD and T-score, the study population was divided into three groups based on serum calcium terciles: low, medium, and high. The second tercile was used as the reference category, as a completely linear association was not observed, in line with findings from other authors [3]. For these statistical tests, we used Statistical Software for Data Science version 18 (STATA v18.0, TX, USA). *p* values < 0.05 are considered statistically significant.

Two approaches were applied for graphical exploration of the association between serum calcium levels and BMD. First, a linear regression model was used to visualize the relationship under the assumption of linearity. Second, a locally weighted scatterplot smoothing (LOWESS) approach was applied to assess potential non-linear trends. These graphical analyses were conducted using R software (version 4.4.1) and were not adjusted for covariates. Additionally, a threshold effect analysis was conducted to explore the relationship between serum calcium levels and BMD at various skeletal sites, applying piecewise linear regression in R. This approach enabled the detection of potential inflection points in the relationship between serum calcium and BMD at different threshold values across the studied sites.

## 3. Results

### 3.1. Characteristics of the Study Population

A total of 966 individuals were included in the study, from which 240 were males, and 726 were females, with a median age of 55 and 59 years, respectively. Females had higher median values than men for body fat percentage, calcium supplementation and vitamin D supplementation. In contrast, male had higher median values than women for waist circumference. Significant differences were also observed in the median BMD of the total hip, lumbar spine, and femur between men and women. The complete characteristics of the study population are shown in Supplementary Table S1.

### 3.2. Minor Allele Frequency of Genetic Variants

**Figure 1:**
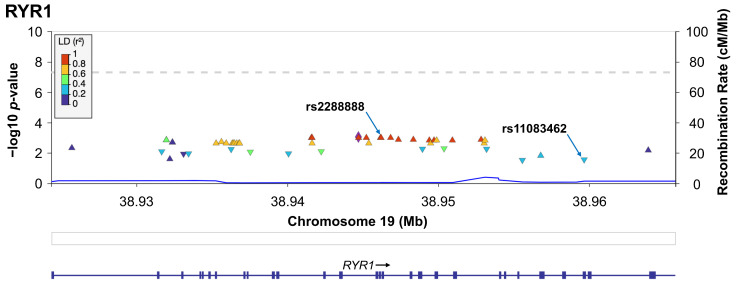
Evidence of association of BMD in the femoral neck with the *RYR1* chromosomal region. The x-axis is the physical position on the chromosome (Mb), and the y-axis denotes the association test result as the −log (*p* value). Genetic variants are color-coded by linkage disequilibrium (LD), arrows show the variants selected for genotyping in the total sample. Regional association plots in the Morelos cohort were generated using LocusZoom, and only variants genotyped from the pilot GWAS are shown.

The synonymous variants rs2288888 (G>A) and rs11083462 (C>T) in *RYR1* were selected for genotyping, based on their previous association with BMD demonstrated through a GWAS in Mexican-mestizo population (*p*-value < 0.05, and Linkage Disequilibrium (LD) (r^2^ ≤ 0.8), in the Morelos population) [22], and a minor allele frequency (MAF) > 5% in the Morelos, MXL (Mexican Ancestry in Los Angeles, CA, USA) and European populations, (Figure 1). Both variants were in Hardy-Weinberg equilibrium, with *p*-values of 0.070 for rs2288888, and 0.713 for rs11083462. The minor allele frequencies (MAF) for variants in our population, were similar to those reported for the Mexican Ancestry population living in Los Angeles, CA, USA (MXL), and European populations in the 1000 Genomes project (Supplementary Table S2).

Supplementary Figure S1 shows localization of the codons harboring the variants in the polypeptide. The rs11083462 lies in codon 1152 corresponding to the SPRY2 domain (Supplementary Figure S1b). The rs2288888 variant belongs to codon 556, corresponding to the subdomain C of the N-terminal domain (Supplementary Figure S1c). Both variants are synonymous, meaning they do not change the amino acid in the protein.

### 3.3. Association Analyses Between Serum Calcium Concentrations and BMD

**Figure 2:**
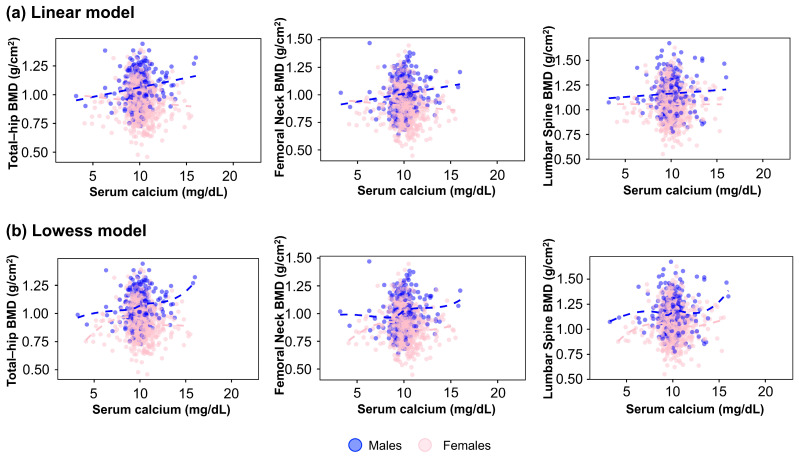
Association between serum calcium levels and bone mineral density (BMD) at the total hip, femoral neck, and lumbar spine. (**a**) Linear regression model. (**b**) LOWESS model. Males (blue dots). Females (pink dots). These models are unadjusted. Dashed line represents the fitted line from the Linear Regression or LOWESS model.

Figure 2 depicts the association between serum calcium levels and BMD at the total hip, femoral neck, and lumbar spine in males and females. A linear regression model was applied in the first row (Figure 2a), showing a weak positive association across all sites, mainly in males. However, when a locally weighted scatterplot smoothing (LOWESS) approach was used (Figure 2b), the relationship appeared non-linear, suggesting potential threshold effects or other underlying patterns not captured by a simple linear model. These findings indicate that assuming linearity may not be appropriate when analyzing the relationship between serum calcium concentration and BMD.

The threshold effect analysis of serum calcium levels on total hip BMD revealed distinct association across sex and age groups. In females, serum calcium levels < 8.5 mg/dL were positively associated with total hip BMD (β = 0.031, 95% CI: 0.003, 0.059), whereas levels above this threshold exhibited an inverse relationship (β = −0.038, 95% CI: −0.067, −0.009) in the fully adjusted model. A similar trend was observed in younger females (<47 years), where higher serum calcium levels (>9.4 mg/dL) was significantly associated with lower total hip BMD (β = −0.079, 95% CI: −0.141, −0.017). In contrast, no statistically significant associations were found in males and females ≥47 years old across serum calcium thresholds (Supplementary Table S3). A comparable pattern was observed for femoral neck BMD, in younger females (<47 years old), serum calcium levels > 9.4 mg/dL were significantly associated with lower femoral neck BMD (β = −0.080, 95% CI: −0.143, −0.018).

Afterwards, we further analyzed the association using serum calcium categorized into tertiles, assessing its relationship with BMD as a continuous variable (linear regression) and low-BMD (logistic regression), using the middle tertile as the reference. This approach allowed us to evaluate potential non-linear effects that may not have been evident in traditional linear modeling. In the linear regression models, females in the highest serum calcium category showed association with the lower total hip (β = −0.024, 95%CI −0.045, −0.003) and femoral neck BMD (β = −0.025, 95%CI −0.045, −0.004) compared to those in the middle category (Table 1).

In the logistic regression models, males in the highest serum calcium category had lower odds of suffering low hip BMD (OR = 0.34, 95%CI 0.12–0.93), while female overall (OR = 1.78; 95%CI 1.10–2.85) and females ≥ 47 years (OR = 1.78; 95%CI 1.10–2.85) had higher odds (Table 2). For low femoral neck BMD, only males in the lowest serum calcium category were at higher odds than those in the middle category (OR = 2.48, 95%CI 1.17–5.27), with no significant associations found in females (Table 2).

### 3.4. Analysis of the Association Between RYR1 Variants, Serum Calcium Concentration and BMD

**Figure 3:**
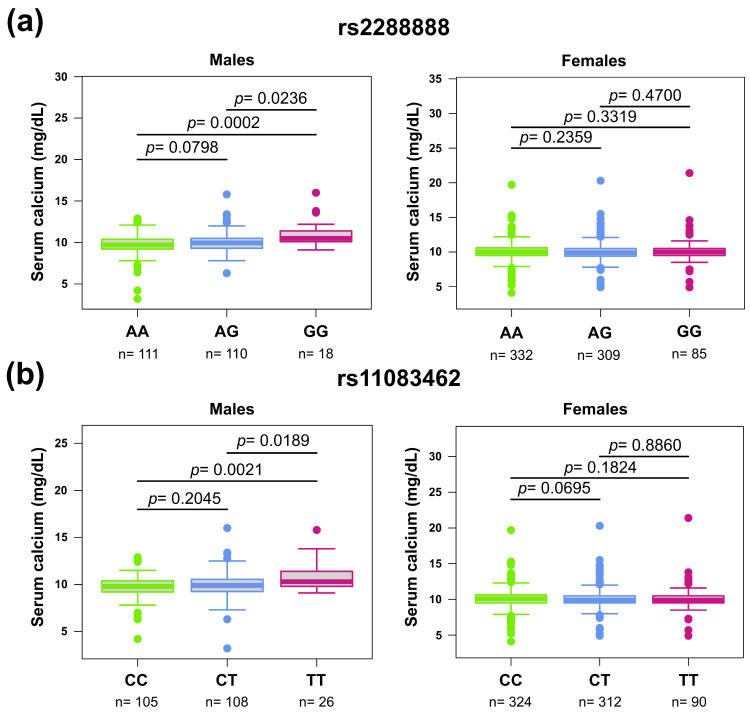
Box plots of serum calcium levels by the genotypes of rs2288888 and rs11083462. (**a**) Differences of serum calcium levels between males and females and genotypes of rs2288888. (**b**) Differences of serum calcium levels between males and females and genotypes of rs11083462. Graphs show median, first and third quartiles. Dots denote observations outside the range of value. *p*-values are from Dunn’s test.

In the total population, homozygous individuals for the G allele of rs2288888 had higher median serum calcium concentrations compared to homozygous for the A allele (10.38 vs. 9.7 mg/dL, *p* = 0.015) (Supplementary Table S4). However, after adjusting for potential confounders, these associations remained statistically significant only in men (Figure 3, Supplementary Table S5). In descriptive analyses, we observed a lower median BMD at total hip and femoral neck in individuals with two copies of the G risk allele of rs2288888 and a lower lumbar spine BMD in those carrying the T allele of rs11083462, compared to the wild-type genotypes (Supplementary Table S4). However, when stratified by sex and adjusted for potential confounders, these associations were no longer significant.

## 4. Discussion

Our findings highlight the significant association between serum calcium concentrations and BMD in specific sex and age groups, with evidence suggesting that the relationship is not linear. These results indicate that the impact of calcium on bone health may be more complex than previously recognized, particularly in women and older women, and warrant further investigation into potential threshold effects and non-linear patterns in bone metabolism. This finding is consistent with a cross-sectional study by Liu M, et al., which revealed a negative association between serum calcium concentration and lumbar spine BMD in elderly women [3]. Additionally, a longitudinal study revealed that women with elevated baseline calcium concentrations exhibited a decreased BMD over ten years, further suggesting a potential role of serum calcium in the development of OP [27]. A series of Mendelian randomization studies in cohorts from the UK, USA, Europe, Australia, and China have demonstrated that elevated serum calcium concentrations do not enhance BMD in the general population [4] nor prevent fracture risk [5]. Furthermore, in adults older than 60 years, such levels may even result in a reduction of BMD [4]. However, the results are controversial, which could be due to the fact that factors such as sex, age and genetics play a crucial role in the relationship between serum calcium concentrations and BMD. These variables may influence how calcium affects bone health, suggesting that the impact of calcium on BMD is more complex and mediated by demographic characteristics, such as sex and age, making it necessary to conduct further analysis in specific populations.

A protective effect on BMD was observed in the male population with the highest serum calcium category. Previous studies have suggested that the association between serum calcium and BMD in males follows a U-shaped curve. For instance, in older men, lumbar BMD increased with serum calcium up to the inflection point at 9.6 mg/dL in a fully adjusted model [3]. In addition to serum calcium concentration, other factors, such as variations in femoral bone composition, could contribute to the risk of low BMD at the femoral neck [28]. Specifically, in USA population, men exhibit lower volumetric bone mineral density (both trabecular and cortical) than women, yet they demonstrate a greater cross-sectional area [29]. Nevertheless, this data should be taken with caution, as a larger and more diverse population is needed to confirm or discard these findings.

Some studies have shown that certain genetic variants are associated with serum calcium concentrations and BMD, for example variants in the vitamin D and calcium-sensing receptor genes [30,31]. In this regard, we investigated whether genetic variants in *RYR1* are associated with serum calcium concentration based on a previous study suggesting that *RYR1* variants could be associated with low BMD [10]. *RYR1* variants can lead to defective calcium release in malignant hyperthermia, myopathies, and neuromuscular disorders [32]. However, the involvement of *RYR1* variants in bone metabolism remains to be elucidated. Nonetheless, patients afflicted with *RYR*-related disorders have been observed to experience skeletal problems, including scoliosis [19] and osteoporosis at an early age [20].

In our study, the variants rs2288888 (g.38455542G>A) and rs11083462 (g.38469040C>T) were associated with higher serum calcium concentrations (under-adjusted and unadjusted data) in males but not females. These findings suggest that the protective effect of calcium concentrations on BMD may partially be attributed to these genetic variants in the male population. In females, these alleles were not associated with calcium concentrations, while higher calcium concentrations were associated with a greater risk of low BMD. These results highlight the role of genetic factors in modulating the relationship between calcium and bone health, particularly between sexes. In addition, the association of genetic variants in the *RYR1* gene with serum calcium could be related to chance. Therefore, additional studies in other populations are needed.

Understanding the effect of serum calcium on BMD is crucial for suggesting strategies for managing OP. Bone mineralization is a complex process in which calcium plays a key role. It regulates osteoblast differentiation and osteoclast activity [33,34]. During osteoclast differentiation and activation, extracellular calcium influx induces calcium release from the endoplasmic reticulum membrane where RYR receptors are located. RANKL/RANK signaling transactivates phospholipase C (PLC) to produce IP3, which binds to inositol 1,4,5-trisphosphate receptors (IP3Rs) on the ER, triggering Ca2+ oscillations [33,34]. Studies have reported that mutations or alterations in the SPRY2 domains and the RHI domain at the N-terminal domain alter RYRs channel function [13,16,35,36]. Despite the fact that the two variants analyzed are found in these domains, it is important to note that the variants studied are synonymous and therefore do not involve a change in the amino acid sequence of the protein.

Even though, the rs2288888 (g.38455542G>A) and rs11083462 (g.38469040C>T) variants are synonymous SNVs, they may affect RYR1 calcium channel activity. Synonymous variants can affect protein translation at different levels, for instance, they can alter the global stability of mRNA, thereby favoring the rapid degradation of less stable mRNA or affecting the stability in the vicinity of the translation start codon [37]. Synonymous variants can also alter the timing between translation and protein folding, affecting protein levels and function [38]. This is particularly important for transmembrane proteins such as membrane receptors, pumps, and channels. Evidence has demonstrated that a ‘silent’ nucleotide change may have clinical implications. A synonymous variant (c.C3435T) in the ATP-driven efflux pump gene MDR1 alters substrate specificity [39], and patients with metastatic renal cell cancer who carried the TCG genotype had unusually prolonged survival when treated with sunitinib due to an alteration in the specificity [40]. To corroborate our hypotheses regarding the potential impact of synonymous genetic variants, further research is necessary. In the future, the objective is to collect peripheral blood samples to evaluate the functional characterization of this, because it has been reported that lymphocytes are a suitable model for this type of study since they express RYR1 [41].

To the best of our knowledge, this is the first report of association between genetic variants in the *RYR1* gene and serum calcium concentrations and BMD in Mexican population. This gene and its respective variants had not been previously linked to bone metabolism, and no reports on their functional roles are available. Although our findings provide new evidence for a previously undescribed association of *RYR1* variants with BMD, some limitations should be considered when interpretating these results. First, the sample size (*n* = 966 individuals) and the limited statistical power for the male population due to the sample size. Second, although we considered potential confounders, residual confounding (like PTH), and population stratification, bias may still exist. However, the MAFs observed in our study were similar to the Europeans and Mexican population living in Los Angeles; therefore, we consider there is little possibility of population stratification. Third, the cross-sectional design of our study limited the ability to establish definitive causal relationships between the investigated variables. While it’s true that genotypes occurred prior to BMD phenotype and serum calcium concentrations, the lack of complete knowledge about the function of genetic variants can hinder the identification of causality. Additionally, we recognize that the association observed between *RYR1* variants and serum calcium concentrations needs to be validated in other independent populations. Functional studies are crucial to determine whether serum calcium concentrations affect the impact of *RYR1* variants on BMD. Despite these limitations, this study contributes with novel knowledge, adding information to the vast number of genes associated with serum calcium in the Mexican population.

## 5. Conclusions

In conclusion, our results highlight significant association between serum calcium concentrations and BMD. However, the relationship was not linear and varied by sex. In women, higher calcium levels were associated with an increased risk of low BMD, while in males, this relationship appeared protective. Additionally, in males, carriers of specific alleles of the rs2288888 and rs11083462 variants, exhibited higher calcium concentrations, suggesting that these genetic variants may influence bone metabolism. These findings indicate that the impact of calcium on bone health is complex and multifactorial, warranting further research to understand threshold and non-linear effects in bone metabolism.

## Figures and Tables

**Table 1:** Association serum calcium categories and BMD of total hip and femoral neck.

			Females	
	Males (*n* = 240)	Females (*n* = 726)	<47 Years (*n* = 143)	≥47 Years (*n* = 583)
	β (95%CI)	β (95%CI)	β (95%CI)	β (95%CI)
Total hip				
Serum calcium categories	Adjusted Model			
Low category	−0.030 (−0.073, 0.014)	−0.008 (−0.028, 0.012)	0.006 (−0.041, 0.053)	−0.005 (−0.028, 0.017)
Medium category	Reference	Reference	Reference	Reference
High category	−0.0003 (−0.047, 0.046)	−0.024 (−0.045, −0.003) *	−0.016 (−0.064, 0.032)	−0.019 (−0.044, 0.005)
Femoral neck				
Serum calcium categories				
Low category	−0.045 (−0.089, 0.002)	−0.007 (−0.027, 0.013)	0.007 (−0.042, 0.056)	−0.004 (−0.026, 0.018)
Medium category	Reference	Reference	Reference	Reference
High category	−0.009 (−0.058, 0.040)	−0.025 (−0.045, −0.004) *	−0.018 (−0.067, 0.032)	−0.018 (−0.042, 0.006)

Model adjusted for age, alcohol consumption, smoking, calcium intake, supplement intake, physical activity, body mass index, hormone replacement therapy (women only), vitamin D levels, and albumin levels. * <0.05. Linear regression models.

**Table 2:** Association between serum calcium categories and BMD of total hip and femoral neck.

			Females	
	Males (*n* = 240)	Females (*n* = 726)	<47 Years (*n* = 143)	≥47 years (*n* = 583)
	OR (95%CI)	OR (95%CI)	OR (95%CI)	OR (95%CI)
Total hip				
Serum calcium categories	Adjusted model			
Low category	1.14 (0.51–2.55)	1.46 (0.92–2.31)	2.59 (0.46–14.45)	1.33 (0.82–2.16)
Medium category	Reference	Reference	Reference	Reference
High category	0.38 (0.14–1.08)	1.78 (1.11–2.87) *	1.62 (0.29–9.15)	1.77 (1.06–2.96) *
Femoral neck				
Serum calcium categories				
Low category	2.48 (1.17–5.27) *	1.14 (0.73–1.79)	0.99 (0.22–4.36)	1.16 (0.72–1.89)
Medium category	Reference	Reference	Reference	Reference
High category	0.99 (0.42–2.33)	1.18 (0.74–1.90)	0.67 (0.15–3.08)	1.30 (0.78–2.18)

Model adjusted for age, alcohol consumption, smoking, calcium intake, supplement intake, physical activity, body mass index, hormone replacement therapy (women only), vitamin D levels, and albumin levels. * <0.05. Linear regression models.

## Data Availability

The data presented in this study are available on request from the corresponding author due to privacy and ethical restrictions.

## Supplementary Materials

The following supporting information can be downloaded at: https://www.mdpi.com/article/10.3390/medsci13040324/s1, Figure S1: Homotetrameric structure of the ryanodine receptor 1 (RyR1) and recurrent variants site rs11083462 (g.38469040C>T) and rs2288888 (g.38455542G>A). Table S1: Clinical characteristics of the study population, Table S2: Genotype/allele frequencies and Hardy-Weinberg Equilibrium (HWE) *p*-values for the genetic variants analyzed, Table S3: Threshold effect analysis of serum calcium on lumbar bone mineral density by using piecewise linear regression, Table S4: Association between the SNVs rs2288888 (g.38455542G>A) and rs11083462 (g.38469040C>T) with variables of interest, Table S5: Association between the genetic variants and serum calcium levels.

## Abbreviations

The following abbreviations are used in this manuscript:

RYR1	Ryanodine Receptor 1
OP	Osteoporosis
BMD	Bone Mineral Density
Ca^2+^	Calcium
