# Supplementary material for: Serum Calcium Concentration Is Associated with Bone Mineral Density and Synonymous Variants in the RYR1 Gene in a Mexican-Mestizo Population
Source: Med Sci (Basel). 2025 Dec 17;13(4):324. doi: 10.3390/medsci13040324 (PMC12734950; doi:10.3390/medsci13040324)
Supplement: Supplementary file 1 [file medsci-13-00324-s001.zip › Supplementary Figure1.pdf]

Serum calcium concentration is associated with bone mineral density and synonymous variants in the gene *RYR1* in a Mexican-mestizo population

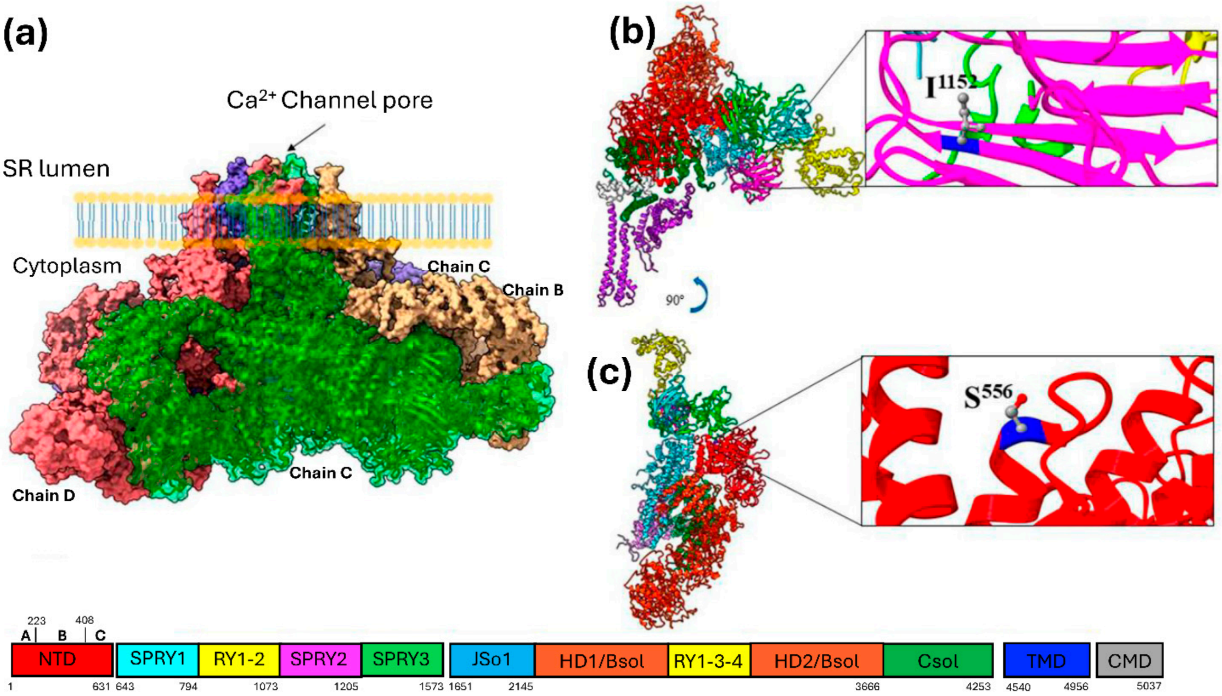

Supplementary Figure 1. Homotetrameric structure of the ryanodine receptor 1 (RyR1) and recurrent variants site rs11083462 (g.38469040C>T) and rs2288888 (g.38455542G>A). (a) Cryo-electron microscopy (cryo-EM) structure of RyR1 in the closed conformation. The cytoplasmic region comprises several domains that serve as scaffolding sites for interactions with activity modulators, such as Ca^{2+} (K3895, E967, E3893, T3966, T5001), caffeine (Q4246, I4996, W4716), ATP (K4214, K4211, H4983, R4215), ryanodine, 2,2′,3,5′,6-pentachlorobiphenyl, the dihydropyridine receptor (DHPR), and the scorpion toxin Imperatoxin-A (IpCa). (b) Enlarged view of the Sprouty RTK Signaling Antagonist 2 (SPRY2) domain highlighting the amino acid containing the SNV rs11083462, Ile1152, encoded within the 38469040 region of the RYR1 gene. The SPRY2 domain is one of three SPRY domains present in each RyR1 subunit. Evidence suggests that the amino-terminal region of this domain interacts with loops II and III of DHPR and with IpCa to modulate channel gating. (c) The SNV rs2288888 corresponds to the S556 residue located within subdomain C of the N-terminal domain (NTD). Mutations in the three NTD subdomains have been associated with diseases such as malignant hyperthermia (MH) and central core disease (CCD). This figure was generated using UCSF ChimeraX with the 7TDG PDB structure as a model. (d) RYR1 protein domains.
