# Supplementary material for: Serum Calcium Concentration Is Associated with Bone Mineral Density and Synonymous Variants in the RYR1 Gene in a Mexican-Mestizo Population
Source: Med Sci (Basel). 2025 Dec 17;13(4):324. doi: 10.3390/medsci13040324 (PMC12734950; doi:10.3390/medsci13040324)
Supplement: Supplementary file 1 [file medsci-13-00324-s001.zip › Supplementary Tables S1-S5.pdf]

Serum calcium concentration is associated with bone mineral density and synonymous variants in the gene *RYR1* in a Mexican-mestizo population

Table S1. Clinical characteristics of the study population.

	**Total**	**Males**	**Females**	
**Variable**	**n=966**	**n=240**	**n=726**	***p*-value**
Age (years)	58.0 (48.0-67.0)	55.0 (46.0-63.0)	59.0 (49.0-68.0)	< 0.001**
BMI (kg/m^2)	26.9 (24.2-30.3)	26.4 (24.2-29.9)	26.9 (24.1-30.7)	0.3477
**Nutritional status, n(%)**				
Overweight	390 (40.4)	95 (39.6)	295 (40.6)	0.7737
Obesity	259 (26.8)	60 (25.0)	199 (27.4)	0.4649
Body fat proportion	43.0 (36.3-47.6)	33.1 (29.0-36.4)	45.3 (40.9-49.6)	<0.001**
Waist circumference (cm)	93.0 (86.0-100.0)	98.0 (91.0-105.0)	91.0 (84.0-99.0)	<0.001**
Serum 25(OH)D levels (ng/ml)	24.8 (19.8-30.0)	25.9 (19.9-30.1)	24.6 (19.8-29.9)	0.1828
Calcium serum (mg/dL)	10.0 (9.4-10.6)	9.9 (9.3-10.6)	10.0 (9.5-10.6)	0.1089
Albumin (mg/dL)	3.9(4.1-4.4)	3.95(4.2-4.4)	3.9(4.1-4.3)	0.0035*
Total hip BMD (g/cm^2)	0.962 (0.865-1.068)	1.053 (0.961-1.171)	0.933 (0.836-1.038)	<0.001**
Lumbar spine BMD (g/cm^2)	1.072 (0.958-1.191)	1.139 (1.035-1.284)	1.046 (0.935-1.163)	<0.001**
Femoral neck BMD (g/cm^2)	0.923 (0.816-1.026)	0.997 (0.901-1.139)	0.894 (0.801-0.996)	<0.001**
T-score, total hip	-0.5 (-1.2-0.3)	-0.3 (-1.0-0.5)	-0.6 (-1.4-0.2)	0.0004**
T-score, lumbar spine	-1.2 (-2.1--0.2)	-0.8 (-1.6-0.2)	-1.3 (-2.2--0.3)	<0.001**
T-score, femoral neck	-0.9 (-1.6--0.1)	-0.5 (-1.3-0.5)	-1.0 (-1.7--0.3)	<0.001**
Vitamin D intake (UI/day)	110.7 (69.2-166.7)	112.2 (77.7-164.8)	110.3 (66.3-168.0)	0.3466
Calcium intake (mg/day)	807.1 (575.6-1124.9)	788.7 (552.2-1044.7)	815.7 (585.0-1177.8)	0.1492
Energy intake (kcal/day)	1755.8 (1307.2-2308.3)	1835.0 (1346.3-2290.5)	1738.2 (1297.2-2312.8)	0.6103
Alcohol intake (g/day)	0.5 (0.2-1.6)	1.4 (0.5-3.6)	0.5 (0.0-1.4)	<0.001**

Calcium supplementation, n(%)	122 (12.6)	0 (0.0)	122 (16.8)	<0.001**
Vitamin D supplementation, n (%)	84 (8.7)	12 (5.0)	72 (9.9)	0.019*
HRT, n (%)	22 (2.3)	0 (0.0)	22 (3.0)	0.002*

Median (P25-P75) for continuous variables. BMI: body mass index, BMD: bone mineral density, HRT: Hormone replacement therapy. * $p < 0.05$, ** < 0.001.

Table S2. Genotype/allele frequencies and Hardy-Weinberg Equilibrium (HWE) *p*-values for the variants analyzed.

				**Minor allele frequency of genetic variants**				
**Variant ID**	**Genotype**	**N**	**%**	**HWCS**	**MXL**	**CEU**	**BEB**	**HWE**
	A/A	443	45.90					
	A/G	419	43.42					
rs2288888	G/G	103	10.68	0.323 (G)	0.281 (G)	0.333 (G)	0.355 (A)	0.070
(g.38455542G>A)	A	1305	67.62					
	G	625	32.38					
	C/C	429	44.46					
	C/T	420	43.50					
rs11083462	T/T	116	12.04	0.337 (T)	0.352 (T)	0.338 (T)	0.424 (C)	0.713
(g.38469040C>T)	C	1278	66.21					
	T	652	33.79					

HWCS: Health Worker Cohort Study, MXL: Mexican ancestry in Los Angeles, California, CEU: Utah Residents with northern and western European ancestry, BEB: Bengali in Bangladesh.

Table S3. Threshold effect analysis of serum calcium on lumbar bone mineral density by using piecewise linear regression.

			Total Hip g/cm^2				
Model 1					Females		
Males (n=240)		Females (n=726)		< 47 years (n=143)		≥ 47 years (n=583)	
	β (95%CI)		β (95%CI)		β (95%CI)		β (95%CI)
Serum calcium < 10.7 (mg/dL)	0.011(-0.008,0.031)	Serum calcium <8.5 (mg/dL)	**0.029(0.001,0.058)**	Serum calcium <9.4 (mg/dL)	0.054 (-0.004, 0.111)	Serum calcium <8.5 (mg/dL)	0.025(-0.009,0.058)
Serum calcium > 10.7 (mg/dL)	0.007(-0.032,0.047)	Serum calcium > 8.5(mg/dL)	**-0.036(-0.065,-0.007)**	Serum calcium >9.4 (mg/dL)	**-0.074 (-0.136, -0.012)**	Serum calcium > 8.5 (mg/dL)	-0.030(-0.064,0.005)
Model 2							
Serum calcium < 10.7 (mg/dL)	0.009(-0.008, 0.0259)	Serán calcium <8.5 (mg/dL)	**0.030(0.002,0.059)**	Serum calcium <9.4 (mg/dL)	0.058 (-0.00009, 0.115)	Serum calcium <8.5 (mg/dL)	0.027(-0.007,0.060)
Serum calcium > 10.7 (mg/dL)	0.014(-0.081,0.109)	Serum calcium > 8.5(mg/dL)	**-0.037(-0.066,-0.008)**	Serum calcium >9.4 (mg/dL)	**-0.077 (-0.140, -0.015)**	Serum calcium > 8.5 (mg/dL)	-0.032(-0.066,0.003)
Model 3							
Serum calcium < 10.7 (mg/dL)	0.009(-0.008,0.026)	Serum calcium <8.5 (mg/dL)	**0.031(0.003,0.059)**	Serum calcium <9.4 (mg/dL)	0.057 (-0.0003, 0.115)	Serum calcium <8.5 (mg/dL)	0.027(-0.006,0.061)
Serum calcium > 10.7 (mg/dL)	0.013(-0.08,0.109)	Serum calcium > 8.5(mg/dL)	**-0.038(-0.067,-0.009)**	Serum calcium >9.4 (mg/dL)	**-0.079 (-0.141, -0.017)**	Serum calcium > 8.5 (mg/dL)	-0.033(-0.067,0.002)

Model 1 adjusted for age, alcohol consumption, smoking status, calcium intake, calcium supplement consumption, physical activity, body mass index, and hormone replacement therapy (only females). **Model 2**: Model 1 + 25(OH)D levels. **Model 3**: Model 2 + albumin levels.

Table S4. Association between the SNVs rs2288888 (g.38455542G>A) and rs11083462 (g.38469040C>T) with variables of interest.

rs2288888	AA (n=443)	AG (n=419)	GG (n=103)	AA vs AG	AA vs GG	AG vs GG
Age (years)	58.0 (48.0-67.0)	59.0 (48.0-67.0)	57.0 (48.0-67.0)	0.4675	0.3483	0.368
BMI (kg/m^2)	26.9 (24.0-30.2)	26.9 (24.3-30.7)	26.7 (24.0-29.5)	0.465	0.133	0.1465
Serum 25(OH)D levels (ng/ml)	25.0 (19.8-29.9)	24.5 (19.6-30.1)	25.1 (19.8-29.9)	0.306	0.4501	0.4242
Calcium serum (mg/dl)	9.7 (9.4-10.6)	9.9 (9.4-10.5)	10.38 (9.5-10.7)	0.4373	**0.015***	**0.0197***
Total hip BMD (g/cm^2)	0.956 (0.863-1.075)	0.975 (0.877-1.068)	0.931 (0.851-1.054)	0.1994	0.0952	**0.034***
Lumbar spine BMD (g/cm^2)	1.074 (0.955-1.216)	1.082 (0.962-1.182)	1.034 (0.952-1.177)	0.4471	0.0514	0.0618
Femoral neck BMD (g/cm^2)	0.924 (0.814-1.028)	0.928 (0.821-1.028)	0.890 (0.812-1.004)	0.2785	0.0825	**0.0405***
T-score, total hip	-0.5 (-1.3,0.3)	-0.5 (-1.2,0.3)	-0.7 (-1.4,0.1)	0.2398	0.1455	0.0684
T-score, lumbar spine	-1.1 (-2.1,-0.1)	-1.1 (-2.0,-0.2)	-1.3 (-2.1,-0.2)	0.3764	0.0753	0.1085
T-score, femoral neck	-0.9 (-1.6,-0.1)	-0.9 (-1.6,-0.1)	-1.1 (-1.7,-0.4)	0.3232	0.0835	**0.0485***
Vitamin D intake (UI/day)	108.1 (65.4-166.9)	111.8 (71.7-166.6)	113.9 (77.7-166.7)	0.1595	0.0888	0.2314
Calcium intake (mg/day)	784.1 (561.7-1107.9)	834.6 (596.1-1130.8)	814.2 (566.0-1178.5)	0.0812	0.1152	0.3661
Energy intake (kcal/day)	1767.4 (1291.4-2293.1)	1781.1 (1327.0-2309.0)	1672.7 (1294.4-2352.0)	0.427	0.354	0.3972
Alcohol intake (g/day)	0.5 (0.2-1.6)	0.5 (0.1-1.6)	0.6 (0.2-1.9)	0.3751	0.1756	0.2317

Calcium supplementation, n (%)	57 (12.9)	50 (11.9)	15 (14.6)	0.6778	0.6467	0.4689
Vitamin D supplementation, n (%)	37 (8.4)	38 (9.1)	9 (8.7)	0.7089	0.899	0.9162
HRT, n (%)	11 (2.5)	8 (1.9)	3 (2.9)	0.5663	0.8038	0.5253
**rs11083462**	**CC (n=429)**	**CT (n=420)**	**TT (n=116)**	**CC vs CT**	**CC vs TT**	**CT vs TT**
Age (years)	58.0 (48.0-68.0)	58.0 (48.0-66.0)	59.5 (49.0-68.0)	0.2674	0.2786	0.1606
BMI (kg/m^2)	26.6 (23.9-30.1)	27.0 (24.2-30.2)	27.5 (24.8-31.4)	0.2168	0.066	0.161
Serum 25(OH)D levels (ng/ml)	25.1 (19.8-29.9)	24.6 (20.0-30.1)	24.5 (19.6-29.8)	0.4989	0.2468	0.2467
Calcium serum (mg/dl)	10.0 (9.4-10.6)	9.9 (9.4-10.5)	10.0 (9.5-10.6)	0.2115	0.2358	0.107
Total hip BMD (g/cm^2)	1.0 (0.9-1.1)	1.0 (0.9-1.1)	0.9 (0.9-1.1)	0.2642	0.2875	0.1655
Lumbar spine BMD (g/cm^2)	1.1 (1.0-1.2)	1.1 (1.0-1.2)	1.0 (0.9-1.2)	0.4711	**0.0492***	**0.045***
Femoral neck BMD (g/cm^2)	0.9 (0.8-1.0)	0.9 (0.8-1.0)	0.9 (0.8-1.0)	0.2175	0.2156	0.0975
T-score, total hip	-0.6 (-1.3-0.3)	-0.5 (-1.2-0.3)	-0.6 (-1.3-0.1)	0.2932	0.2945	0.1853
T-score, lumbar spine	-1.1 (-2.1--0.1)	-1.1 (-2.0--0.2)	-1.4 (-2.2--0.2)	0.4874	0.0504	**0.0487***
T-score, femoral neck	-0.9 (-1.6--0.1)	-0.8 (-1.6--0.1)	-1.1 (-1.6--0.3)	0.2308	0.1942	0.0896
Vitamin D intake (UI/day)	105.9 (64.2-158.9)	116.0 (74.8-171.8)	109.0 (71.5-167.7)	**0.0021***	0.0837	0.314
Calcium intake (mg/day)	763.6 (552.3-1065.6)	850.6 (611.6-1169.6)	815.2 (573.5-1190.1)	**0.0077***	**0.0444***	0.4502
Energy intake (kcal/day)	1737.0 (1267.9-2290.5)	1781.1 (1337.0-2306.2)	1772.1 (1302.3-2383.1)	0.2982	0.1691	0.2698
Alcohol intake (g/day)	0.5 (0.2-1.5)	0.5 (0.2-1.9)	0.5 (0.2-1.6)	0.2516	0.4664	0.3014
Calcium supplementation, n(%)	54 (12.6)	53 (12.6)	15 (12.9)	0.9889	0.9213	0.9288
Vitamin D supplementation, n(%)	37 (8.6)	41 (9.8)	6 (5.2)	0.5663	0.2211	0.1219

HRT. n(%)	10 (2.3)	10 (2.4)	2 (1.7)	0.9617	0.6927	0.6721

BMI: body mass index, BMD: bone mineral density, HRT: Hormone replacement therapy. * $p < 0.05$.

Table S5. Association between the genetic variants and serum calcium levels.

rs2288888	Males		Females	
**Model**	**β(95%CI)**	***p*-value**	**β(95%CI)**	***p*-value**
Additive	0.034(0.006,0.063)	0.017	-0.002(-0.017,0.014)	0.844
AA	Ref		Ref	
AG	0.017(-0.023,0.057)	0.397	-0.003(-0.025,0.020)	0.822
GG	0.086(0.023,0.149)	**0.008**	-0.002(-0.036,0.020)	0.899
AA	Ref		Ref	
AG+GG	0.031(-0.007,0.069)	0.108	-0.002(-0.023,0.018)	0.817
AA+AG	Ref		Ref	
GG	0.078(0.018,0.138)	**0.011**	-0.0009(-0.033,0.031)	0.954
rs11083462				
Additive	0.041(0.011,0.071)	0.007	0.004(-0.013,0.018)	0.759
CC	Ref		Ref	
CT	0.033(-0.007,0.072)	0.102	-0.0002(-0.022,0.022)	0.982
TT	0.100(-0.022,0.171)	**0.011**	0.007(-0.027,0.041)	0.677
CC	Ref		Ref	
CT+TT	0.042(0.004,0.080)	**0.028**	0.001(-0.019,0.022)	0.899
CC+CT	Ref		Ref	
TT	0.082(0.010,0.155)	**0.026**	0.007(-0.025,0.039)	0.655

Serum calcium levels were log-transformed. Model adjusted by age, sex, alcohol intake, calcium intake, calcium supplements, smoking status, vitamin D levels, hormone replacement therapy and body mass index.
